# Awareness and Attitudes of the Pakistani Population With Regard to Physician–Pharmaceutical Company Interaction: A Cross-Sectional Study

**DOI:** 10.3389/fphar.2021.787891

**Published:** 2022-01-07

**Authors:** Ali Hassan Gillani, Sumaira Omer, Hafsa Arshad, Wenchen Liu, Chen Chen, Sadia Bashir, Asma Bashir Ahmed, Abubakar Munir, Amna Saeed, Kamran Bashir, Yu Fang

**Affiliations:** ^1^ Department of Pharmacy Administration and Clinical Pharmacy, School of Pharmacy, Xi’an Jiaotong University, Xi’an, China; ^2^ Center for Drug Safety and Policy Research, Xi’an Jiaotong University, Xi’an, China; ^3^ Centre for Health Reform and Development Research, Xi’an, China; ^4^ Department of Pharmacy, Quaid-i-Azam University, Islamabad, Pakistan; ^5^ Department of Pathology, Quaid E Azam Medical College, Bahawalpur, Pakistan; ^6^ Institute of Pharmaceutical Sciences, Jinnah Sindh Medical University, Karachi, Pakistan; ^7^ Department of Pharmaceutical Sciences, The Superior University, Lahore, Pakistan; ^8^ Department of Pharmaceutical Sciences, Shahida Islam Medical College, Lodhran, Pakistan

**Keywords:** physician, pharmaceutical company, interaction, Pakistan, general public

## Abstract

**Objective:** To determine the awareness and attitudes of the Pakistani population regarding physician–pharmaceutical company interactions.

**Methods:** The data were collected from primary health care clinics and pharmacy outlets located within cities of six randomly selected districts of the Punjab Province. Those individuals (age ≥18 years) who have just completed their visit to the physician and well understand Urdu language were approached. Descriptive analysis was performed for all variables by using SPSS (IBM version 26).

**Results:** A total of 3,852 participants fully completed the study out of 4,301 (response rate 89.5%). Of those, 30.9% were female; two-thirds (66.7%) were aware of drug representatives’ visits to clinics. The majority were aware of pharmaceutical company material presence (or absence) in the physicians’ rooms (56.6%), company items with logos (66.8%), patient education materials (73.4%), and 60.8% thought that receiving gifts from companies was “wrong/unethical” practice for physicians, which was lower in comparison to other professions such as judges to accept gifts from lawyers (65.6%) and professional sports umpires to acknowledge gifts (64.3%). A minority said that they have lower trust on physicians for using drug company notepads or pens (16.7%), going on trips sponsored by the company (16.7%), accepting gifts <15,000 PKR (90.3 US$) (26.7%), and accepting gifts >15,000 PKR (90.3 US$) (40.0%).

**Conclusion:** Survey participants were well aware of physician–pharmaceutical company interactions. Participants were more knowledgeable regarding the pharmaceutical company presence (or absence) in physicians’ offices than about gift-related practices of physicians. Trust on the physician was not affected by small gifts but by the large gifts.

## Introduction

The pharmaceutical industry (PI) uses numerous tactics for promoting its products to physicians such as offering gifts, free meals, and advertisements (King et al., 2013). Although, pharmaceutical manufacturers claim that drug promotional activities are intended for the education of the physician, the accelerated promotion of medicinal products is possibly linked to the prescription behavior (Epstein et al., 2013; King et al., 2013). In high-income countries such as the U.S., all payments used to promote pharmaceutical products must be disclosed under the Sunshine Act. In addition, to maintain the transparency of the interaction between the PI and physician, an open payment database has also been created through which all industry payments to physicians and teaching hospitals can be retrieved (Greenway and Ross, 2017).

The previous literature has highlighted that in a Pakistani health care system, physicians prefer to prescribe selected brand medications over generics, which are usually more costly than their generic counterparts (Sharif et al., 2016). In 2019, a study was conducted to document the interaction between physicians and medical sales representatives in Pakistan. The study found that according to perceptions of medical sale representatives 33% of physicians were indulged in unethical prescribing and 42% never made an inquiry or sought evidence for promoting medicines, whereas 70% of the physicians claimed that medical sales representatives ignored patient’s well-being to achieve their sales objectives (Naqvi et al., 2019).

It is noticeable that the impact of drug promotional activities is not only limited to physicians, but also it has put patient’s health and safety on stake as well. These interactions can also have implications for patient’s safety and care. The irrational prescription based on the promotion of specific brands can bring negative clinical and humanistic outcomes for patients, subsequently lowering general public’s trust toward a health care system (Ammous et al., 2017). In Pakistan, most health care expenditure is out of pocket, and usually poor patients bear the burden of these expensive medicines. Moreover, doubts and lack of trust on health care professionals may reduce levels of patient satisfaction, poor adherence to screening recommendation, and treatment (Safran et al., 1998). In total, patients get lower quality of care, whenever unethical promotional activities take place.

Considering its potential impact on patient’s well-being, many studies have been performed in other countries to determine perceptions of patients toward this relationship (Semin et al., 2006; Green et al., 2012; Ammous et al., 2017). Based on a systematic review of 20 studies, it is already clear that patients have limited knowledge about interactions between physicians and drug companies (Fadlallah et al., 2016). As far as we know, no study has yet been conducted in Pakistan to investigate the perception and knowledge of the general public regarding the physician–pharmaceutical company interaction. Therefore, this is the first study aimed to evaluate the awareness and attitudes of the Pakistani population about the interactions between physicians and pharmaceutical companies.

## Methods

### Study Area

Pakistan is the state of four provinces, two independent territories, and federal capital area. There is a further subdivision of provinces, districts, tehsils, and villages (the lowest level in the demographic pyramid). Punjab covers 26% of the total land area of Pakistan and occupies nine divisions and 36 districts. Also, 60% of Pakistan’s population resides in Punjab^1^. Six districts were chosen randomly from all districts of Punjab Province. From each district, one city was conveniently selected taking into consideration of convenience of the data collectors.

### Study Population and Recruitment

Adult participants (age ≥18 years) who can understand both Urdu and English were recruited in this study. We conveniently approached individuals who just completed the visits to the doctor in private health care clinics. The clinic was selected on the basis of greater patient flow and seniority of the doctor. We also approached the patients from the community pharmacy outlets who had just visited the doctor. We excluded those who worked as personnel at recruitment sites and those who were with patients. Data collectors initially contacted eligible clinics and pharmacies to request permission to conduct interviews with patients. Then, from June 2020 to December 2020, team members visited the clinics, where they approached, and recruited potential participants conveniently. In addition, the team visited pharmacies, where they approached people conveniently and invited them to participate.

### Training of Data Collectors

A total of six teams of data collectors were designated to collect data simultaneously in six different districts of Punjab. A team of two data collectors collected data from each city. They were mostly comprised of the local pharmacy students. A prior training was given which involved the following aspects: presenting a brief introduction of the study purpose to respondents, approaching the candidate, conducting face-to-face interviews, and coping with respondent’s lack of cooperation or other difficulties during the interview. The training was carried out for 3 days, with a demonstration given by the primary researcher. The trainees then conducted a pilot study in each of their respective districts and were observed for their interviewing skills.

### Survey Tool

We took our data collection tool from a previous self-administered, validated questionnaire designed by Ammous et al. (2017). We translated the questionnaire from English to Urdu version and then translated it back to English. The survey included 52 questions including the following domains: demographic characteristics and health care–related questions (*n* = 14); awareness (*n* = 15); attitudes (*n* = 11); beliefs (*n* = 8), and items identifying the influence of the medical representation promotion on the prescription of antibiotics (*n* = 4). Before the start of data collection, a pilot study was conducted in each area to check the accuracy of the wording and comprehensiveness. The data obtained in the pilot study were not included in the final analysis.

### Ethical Approval

This study was approved by the Ethics Committee for Medical Research of Xi’an Jiaotong University (Reference # PROM2021-02) and the Research Ethics Committee of the Pharmacy Department of the Superior University Lahore. Participation in this study was voluntary. Prior verbal approval was obtained from the participants, and all the respondents were made aware of their right to leave the study at any time. No personal identifying information was collected, and they were asked to sign the paper-based questionnaire.

### Data Collection

We collected data from June 2020 to December 2020. The research team visited the waiting areas of clinics and pharmacy outlets and conducted the interview from eligible individuals who consented to participate. The interview was conducted in privacy.

### Data Analysis

We entered the data into SPSS (IBM version 26) and then cross-checked by another one. Descriptive analysis was carried out for all variables. We presented the percentages for demographics in tables for each of three categories (demographics, awareness, and attitudes and beliefs).

## Results

### Demographics and Health-Related Conditions

We invited patients from waiting rooms of 12 primary health care clinics and six pharmacies. Of the 4,301 individuals approached, 3,991 were willing to participate, while 3,852 completed the study (response rate 89.5%). The age range was from 18 to 77 years with mean ± SD age of 35.9 ± 12.8. They were predominantly male (69.1%), and 33.3% had an educational level of college or higher. A large population had incomes between 15,000 and 30,000 PKR (91US$-181 US$; 41.2%), and only 3.5% had incomes over 50,000 PKR (305 US$). Out of the total, 40% were diagnosed with the chronic condition such as diabetes or hypertension, 23.4% were diagnosed with mental health conditions, and 23.3% were patients of stroke or myocardial infarction. 23.4% reported that they have a personnel health care provider, and 57.4% of them were satisfied with them; 30% of the total received the medicine sample in the last 1 year, and 63.3% currently using prescription medications (Table 1).

**TABLE 1:** Demographics and health-related conditions.

Demographic	N (percentage)
**Age (mean ± SD) years**	
35.9 ± 12.8	
**Gender**	
Male	2,660 (69.1)
Female	1,192 (30.9)
**Education**	
Less than high school	1,288 (33.4)
High school to college	1,280 (33.1)
College and higher education	1,284 (33.3)
**Monthly income (PKR)**	
Less than 15,000	1,198 (31.1)
15,000–30,000	1,588 (41.2)
30,001–50,000	932 (24.2)
Greater than 50,000	134 (3.5)
**Employment status**	
Employed for pay	1,602 (41.6)
Self	1,358 (35.3)
Retired	378 (9.8)
Not working/disabled	514 (13.3)
**Diagnosed for the chronic condition such as diabetes or hypertension**	
Yes	1,540 (40.0)
No	2,312 (60.0)
**Diagnosed for mental health condition**	
Yes	900 (23.3)
No	2,952 (76.7)
**Diagnosed for stroke or MI**	
Yes	898 (23.3)
No	2,954 (76.7)
**Do you have a personnel health care provider?**	
Yes	902 (23.4)
No	2,950 (76.6)
**If yes, are you satisfied with the health care provider? (902)**	
Yes	518 (57.4)
No	384 (42.6)
**Are you currently using any prescription medicine?**	
Yes	2,440 (63.3)
No	1,412 (36.7)

### Awareness of Gifts

Table 2 demonstrates the participants’ awareness of PI item’s presence in physicians’ offices. Most of the patients were aware whether or not (answered “yes” or “no” as opposed to “I do not know”) the following items were there in the physician’s room: drug company advertisements (56.6%), company items with logos on them (66.8%), and patient education materials (73.4%). Two-thirds (66.7%) of the respondents were aware of PR’s visit to the clinic, and 53.3% were aware of office staff eating lunches paid by the drug companies.

**TABLE 2:** Awareness of the pharmaceutical company items presence in physicians’ offices (Are the following present in the exam room, waiting room, or other areas of your physician’s’ office?).

Pharmaceutical company item	Yes	No	Do not know
	Number (%)	Number (%)	Number (%)
Drug company advertisement	1,796 (46.6)	386 (10.0)	1,670 (43.4)
Items with the drug company logo	1,930 (50.1)	642 (16.7)	1,280 (33.2)
Office staff eating lunches paid by the drug companies	778 (20.2)	2,048 (53.2)	1,026 (26.6)
Drugs representative presence in the office or waiting room	1,800 (46.7)	770 (20.0)	1,282 (33.3)
Patient education material with the drug logo on it	1,668 (43.3)	386 (10.0)	1798 (46.7)

Table 3 shows the patients’ level of awareness on gift-related practices of physicians. A minority of the population knew whether or not physician went on paid trips by the drug companies (43.4%), accepted large gifts >15,000 PKR [90.3 USD] (33.5%), conducted research for drug companies (32.2%), accepted meals offered by drug companies (33.3%), attended drug company social activities (43.4%), or gave lectures for the drug company (23.4%). However, half of the respondents were aware that the doctor accepted small gifts <15,000 PKR [90.3 USD] (50.0%), and more participants were aware that the physician used drug company pens or notepads (56.6%).

**TABLE 3:** Physician engagement activities with pharmaceutical companies (Knowledge of physician engagement in a variety of activities with pharmaceutical companies. Does your doctor?).

Statement	Yes	No	Do not know	Do not care	Do not know but want to know
Accepting gift <15,000 PKR [90.3 USD]	1,154 (30.0)	770 (20.0)	770 (20.0)	770 (20.0)	388 (10.0)
Attend drug company social activities	1,158 (30.1)	514 (13.3)	1,282 (33.3)	642 (16.7)	256 (6.6)
Go on trips paid by the drug company	514 (13.3)	1,156 (30.0)	898 (23.3)	898 (23.3)	386 (10.0)
Accepting gifts >15,000 PKR [90.3 USD]	648 (16.8)	644 (16.7)	1,408 (36.6)	896 (23.3)	256 (6.6)
Give lecture for the drug company	388 (10.1)	518 (13.4)	2,048 (53.2)	768 (19.9)	130 (3.4)
Conduct research for the drug company	648 (16.8)	516 (13.4)	1,536 (39.9)	512 (13.3)	640 (16.6)
Accept drug company meal	770 (20.0)	512 (13.3)	512 (13.3)	1,026 (26.6)	1,032 (26.8)
Use drug company notepads or pens	1,922 (49.9)	258 (6.7)	256 (6.6)	1,416 (36.8)	0 (0.0)

### Attitudes About Gifts

Table 4 shows the percentage of population who agreed with a series of statements about physicians’ acceptance of gifts or meals and their prescribing behaviors (50.4%); it is ok to accept low-value gifts (64.1%); the practice is wrong/unethical (43.8%); accepting meals makes patients wait too long (59.7%); and this activity is not problematic (60.8%).

**TABLE 4:** Populations view on physicians accepting gifts and their prescribing behavior (What is your attitude toward your physician accepting small gifts or meal?).

Statement	Agree	Neither agree nor disagree	Disagree
It influences my doctor’s prescribing behavior	1,940 (50.4)	1,266 (32.8)	646 (16.8)
It is OK, as long as gifts are of the little monetary value	2,468 (64.1)	994 (25.8)	390 (10.1)
It is not a problem	2,342 (60.8)	1,140 (29.6)	370 (9.6)
It is wrong/unethical	1,686 (43.8)	1,170 (30.4)	996 (25.8)
It makes patients wait too long	2,298 (59.7)	1,298 (33.7)	256 (6.6)
It underestimates my trust in my doctor	1,922 (49.9)	1,284 (33.3)	646 (16.8)

Table 5 shows attitudes of the populations about various professionals accepting meals or small gifts. The response of the population reporting that it was ‘wrong/unethical’ for doctors to accept gifts from drug company representatives (60.8%) was lower from judges to accept gifts from lawyers (65.6%), professional sports umpires to acknowledge gifts from players, whose games they supervise (64.3%), and politicians to take presents from lobbyists (62.5%).

**TABLE 5:** Attitude of the population toward various professionals accepting meals or small gifts (How proper do you think it is for each of the following to accept meals or small gifts from those listed?).

Statement	Not a problem	Neutral	Unethical/wrong
Judges (from lawyers whose case they are hearing)	385 (10.0)	942 (24.5)	2,525 (65.5)
Professional sports referees (from players whose games they officiate)	379 (9.8)	998 (25.9)	2,475 (64.3)
Politicians (from lobbyists)	412 (10.7)	1,033 (26.8)	2,407 (62.5)
Doctors (from drug company representatives)	516 (13.4)	993 (25.8)	2,343 (60.8)
Business people (from clients)	652 (16.9)	1,028 (26.7)	2,172 (56.4)

### Impact on Trust in Physicians

Table 6 reports the number of populations which showed lower trust on the physician in relation to their participation in various promotional activities such as using drug company notepads or pens (16.7%), accepting gifts >15,000 PKR (>92 US$) (40.0%), going on trips sponsored by the PI (16.7%), and accepting gifts <15,000 PKR (<92 US$) (26.7%). However, it is worth mentioning that trust on the physician was more if he/she had a relationship with the PI, i.e., conducting research for the pharmaceutical company (53.3% had an increased trust) and giving lectures for the PI (53.2% had increased trust).

**TABLE 6:** Effect of different promotional activities on physicians’ trust (How will each of the following affect your level of trust in your physician?).

Statement	Higher trust	No change	Lower trust
Accepting gifts <15000 PKR [90.3 USD]	256 (6.6)	2,568 (66.7)	1,028 (26.7)
Attend drug company social activities	1,536 (39.9)	1,544 (40.1)	772 (20.0)
Go on trips paid by the drug company	642 (16.7)	2,566 (66.6)	644 (16.7)
Accept gifts >15000 PKR [90.3 USD]	1,284 (33.3)	1,028 (26.7)	1,540 (40.0)
Give lectures for the drug company	2,050 (53.3)	1,288 (33.4)	514 (13.3)
Conduct research for the drug company	2,052 (53.3)	1,284 (33.3)	516 (13.4)
Accept drug company meal	1,030 (26.7)	1,769 (46.7)	1,026 (26.6)
Use drug company pens or notepads	1,666 (43.3)	1,544 (40.0)	642 (16.7)

### Influence of the Medical Representation Promotion on the Prescription of Antibiotics

Of the total, 51.0% were prescribed with the antibiotics in the recent meeting with the doctor; 60.6% said that drug representatives were affecting physicians to prescribe the antibiotics for the ailments in which the antibiotics were not required, and 63.3% thought that the doctors who received gifts from medical companies prescribed more antibiotics than others (data not shown in tables).

## Discussion

This study explored the awareness and attitudes of the Pakistani general population regarding physician–pharmaceutical company interactions. In this study, most were aware of the presence (or absence) of pharmaceutical companies in physician clinics, with a smaller proportion aware of physician gift practices. Half of the population thought that accepting small gifts or meals by physicians affected the prescribing practices of the doctor, and almost two-thirds of the respondents said drug representatives were affecting physicians to prescribe the antibiotics for those diseases in which it is not required and who received gifts prescribed more antibiotics than others. These results were in line with the previously conducted study in Lebanon, where participants were generally more aware of PI presence (or absence) in physicians’ offices and accepting meals affects the prescribing practices (Ammous et al., 2017).

Some interesting facts highlighted in our study are that level of awareness was high in relation to pharmaceutical company presence (or absence) in doctors’ clinics and that of gift-related practices of physicians, it is somewhat similar with the study in the Lebanon (Ammous et al., 2017). Similar outcomes were observed in developed countries. A study conducted in 2008 in the United States found that 82% of the respondents were aware of the presence of small gifts (pens or notepads) in physicians’ clinics, as compared with 56.6% in our study (Green et al., 2012). The participants who were aware of physicians accepting gifts >15,000 PKR, gifts <15,000 PKR, and trip invitations were, respectively, 12, 16, and 34% in the United States, 29, 31, and 30% in Lebanon, and 33.5, 50.0, and 43.3% in our study. One of the most probable explanations is that the drug company presence in the office is typically noticeable (e.g., drug company advertisements, items, and education material with drug company logos on them), whereas most of other interactions of physicians with drug companies are not. This highlights the need for transparency and disclosure by physicians.

In our study, the number of respondents who believed that gifts and pharmaceutical interactions between physicians and PI affect the physicians’ prescribing pattern was 50.4%, which is slightly higher than in the United States (41%) and Lebanon (44%) (Jastifer and Roberts, 2009; Ammous et al., 2017). Our study also highlighted that 60.8% of the population said that it is unethical for the doctors to accept the gifts from companies; these are in line with results in Turkey, where 71% patients admitted to primary health care centers in 2004 agreed that accepting gifts from the drug companies is not ethical (Semin et al., 2006) and same is in the United States (Blake and Early, 1995). But, these results were much higher than the previous results from Pakistan (9%) (Qidwai et al., 2003). It is evident in our results that some individuals would be opposed to this unethical practice (physicians’ pharmaceutical interaction) and believe it affects physicians’ prescribing behavior (43.8%). Interestingly, the question that demonstrates the ethicality of similar practices (accepting small gifts and meals) with other professions indicated that the respondents might have different standards or expectations across professions. The number of respondents who thought that it is wrong and unethical for doctors to accept was low as compared to referees, judges, and politicians.

In our study, the minority of respondents reported (6.6%) lower trust in response to accepting gifts by the physicians and suggests that relatively few of them made a connection between physicians’ practices and their behaviors. As in other studies, it has been indicated that patients’ lack of trust is associated with the possibility that physicians chose drugs that are more expensive, less efficacious, and cause higher side effects (Jastifer and Roberts, 2009). Notably, a substantial proportion of the participants reported that they had more trust in their physician if he/she had a relationship with PI; it was also observed in Lebanon (Ammous et al., 2017). It is therefore hypothesized that there is a belief that close interaction between the two leads to enhanced physician’s awareness of the newest pharmaceutical innovations, whereas participants of the study conducted in the United States indicated a greater decrease in their level of trust on knowing that their physicians were accepting monetary gifts and enjoying trips paid by drug companies. We also illustrated examining the change in the level of trust when PI offers paid trips to physicians, 66.6% of our population said no change which is quite higher than in the Lebanese population (55%), while 30% reported lower trust. Conversely in the American study sample which showed that 58% reported a decrease in the level of trust in doctors’ accepting paid trips by PI, while 38% had no change in their level of trust (Green et al., 2012). It is a clear-cut indication of the fact that our population is less aware of the potential harm of these interactions than the Lebanese and the American population. As for the comparison, the percentages of people stating lower trust in physicians related to their acceptance of gifts >15,000 PKR [90.3 USD] and to trips paid by the PI were, respectively, 50% and 58% (2008) in the US survey, 45 and 30% in the Lebanese survey, and 33.3 and 16.7% in our sample.

We aimed to target the larger population in the wider area of Punjab. Though the sample is large, our sample is not representative of the general Pakistani population. However, representativeness was enhanced by inclusion of patients (from primary health care clinics) and pharmacy outlets. From a policy perspective, there is a definite need to raise awareness among the Pakistani population about the potential negative impacts of interaction between physician and industry on the cost and quality of their health care. On a broader level, there is a need for system-level interventions to regulate physician–industry interactions (Alkhaled et al., 2014). These interventions should focus governmental and self-regulation (e.g., voluntary codes of practice). The ultimate aim would be to minimize any negative effects of the physician–pharmaceutical company interactions on patients and eventually improve patient care.

The study has some limitations too such as we assessed the knowledge by close-ended questions; respondents may have selected the most favorable answers rather than accurate ones, and therefore a qualitative approach might be more suitable to reveal misconceptions. Also, we selected the districts randomly and targeted the cities and participants from the cities conveniently. In addition, the questionnaire has not been validated. The private clinics with higher patient flows were selected in order to obtain a large sample. It, however, creates the biasness. We recruited patients from the pharmacy sites as well.

## Conclusion

Survey respondents were well aware of the interactions between pharmaceutical companies and doctors. Participants were more knowledgeable regarding the pharmaceutical company presence (or absence) in physicians’ offices than about gift-related practices of physicians. Pharmaceutical interactions were widely believed to affect the way physicians prescribe drugs. Accepting gifts from pharmaceutical companies for doctors is considered less bad/unethical than judges accepting gifts from lawyers and referees from athletes. The trust level was not affected for various types of physician–pharmaceutical company interactions except for receiving large gifts.

## Data Availability

The raw data supporting the conclusion of this article will be made available by the authors, without undue reservation.

## Footnotes

1 Pakistan Bureau of Statistics. (2021). Population Census. https://www.pbs.gov.pk/content/population-census [Accessed September 08, 2021].

## References

[B1] Alkhaled L. Kahale L. Nass H. Brax H. Fadlallah R. Badr K. (2014). Legislative, Educational, Policy and Other Interventions Targeting Physicians' Interaction with Pharmaceutical Companies: a Systematic Review. BMJ Open 4 (7), e004880. 10.1136/bmjopen-2014-004880 PMC4091460 24989618

[B2] Ammous A. Bou Zein Eddine S. Dani A. Dbaibou J. El-asmar J. M. Sadder L. (2017). Awareness and Attitudes of the Lebanese Population with Regard to Physician-Pharmaceutical Company Interaction: a Survey Study. BMJ Open 7 (3), e013041. 10.1136/bmjopen-2016-013041 PMC5387964 28363922

[B3] Blake R. L. Early E. K. (1995). Patients' Attitudes about Gifts to Physicians from Pharmaceutical Companies. J. Am. Board Fam. Pract. 8, 457–464. 8585404

[B4] Epstein A. J. Busch S. H. Busch A. B. Asch D. A. Barry C. L. (2013). Does Exposure to Conflict of Interest Policies in Psychiatry Residency Affect Antidepressant Prescribing. Med. Care 51 (2), 199–203. 10.1097/MLR.0b013e318277eb19 23142772

[B5] Fadlallah R. Nas H. Naamani D. El-jardali F. Hammoura I. Al-khaled L. (2016). Knowledge, Beliefs and Attitudes of Patients and the General Public towards the Interactions of Physicians with the Pharmaceutical and the Device Industry: a Systematic Review. PloS One 11 (8), e0160540. 10.1371/journal.pone.0160540 27556929 PMC4996522

[B6] Green M. J. Masters R. James B. Simmons B. Lehman E. (2012). Do gifts from the Pharmaceutical Industry Affect Trust in Physicians. Fam. Med. 44 (5), 325–331. 23027114

[B7] Greenway T. Ross J. S. (2017). US Drug Marketing: How Does Promotion Correspond with Health Value. BMJ 357, j1855. 10.1136/bmj.j1855 28465309

[B8] Jastifer J. Roberts S. (2009). Patients' Awareness of and Attitudes toward Gifts from Pharmaceutical Companies to Physicians. Int. J. Health Serv. 39 (2), 405–414. 10.2190/HS.39.2.j 19492632

[B9] King M. Essick C. Bearman P. Ross J. S. (2013). Medical School Gift Restriction Policies and Physician Prescribing of Newly Marketed Psychotropic Medications: Difference-In-Differences Analysis. BMJ 346, f264. 10.1136/bmj.f264 23372175 PMC3623604

[B10] Naqvi A. A. Zehra F. Khan N. Ahmad R. Mcgarry K. (2019). Report: Interactions and Conflicts of Interests between Prescribers and Medical Sales Representatives (MSRs) Regarding Prescribing and Drug Promotion Practices in Karachi, Pakistan. Pak. J. Pharm. Sci. 32 (2), 687–695. 31081784

[B11] Qidwai W. Qureshi H. Ali S. S. Alam M. (2003). Perceptions on Bioethics Among Patients Presenting to Family Physicians at a Teaching Hospital in Karachi. Pak. J. Med. Sci. 19 (3), 192–196. 11475782

[B12] Safran D. G. Taira D. A. Rogers W. H. Kosinski M. Ware J. E. Tarlov A. R. (1998). Linking Primary Care Performance to Outcomes of Care. J. Fam. Pract. 47 (3), 213–220. 9752374

[B13] Semin S. Güldal D. Ozçakar N. Mevsim V. (2006). What Patients Think about Promotional Activities of Pharmaceutical Companies in Turkey. Pharm. World Sci. 28 (4), 199–206. 10.1007/s11096-006-9032-8 17066242

[B14] Sharif H. Sharif S. Zaighum I. (2016). Reasons of Brand Switching and Preference in Prescription Medicines: A Comparison between Physicians and Pharmacists of Karachi. Pharm. Innov. 5 (7), 99.

